# Functional homogeneity and specificity of topological modules in human proteome

**DOI:** 10.1186/s12859-018-2549-8

**Published:** 2019-02-04

**Authors:** Rama Kaalia, Jagath C. Rajapakse

**Affiliations:** School of Computer Science and Engineering, Nanyang Technological University, Singapore, Singapore

**Keywords:** Topological modules, Functional modules, Physical PPI, Functional PPI, Functional enrichment analysis, Protein-protein interaction networks

## Abstract

**Background:**

Functional modules in protein-protein interaction networks (PPIN) are defined by maximal sets of functionally associated proteins and are vital to understanding cellular mechanisms and identifying disease associated proteins. Topological modules of the human proteome have been shown to be related to functional modules of PPIN. However, the effects of the weights of interactions between protein pairs and the integration of physical (direct) interactions with functional (indirect expression-based) interactions have not been investigated in the detection of functional modules of the human proteome.

**Results:**

We investigated functional homogeneity and specificity of topological modules of the human proteome and validated them with known biological and disease pathways. Specifically, we determined the effects on functional homogeneity and heterogeneity of topological modules (i) with both physical and functional protein-protein interactions; and (ii) with incorporation of functional similarities between proteins as weights of interactions. With functional enrichment analyses and a novel measure for functional specificity, we evaluated functional relevance and specificity of topological modules of the human proteome.

**Conclusions:**

The topological modules ranked using specificity scores show high enrichment with gene sets of known functions. Physical interactions in PPIN contribute to high specificity of the topological modules of the human proteome whereas functional interactions contribute to high homogeneity of the modules. Weighted networks result in more number of topological modules but did not affect their functional propensity. Modules of human proteome are more homogeneous for molecular functions than biological processes.

**Electronic supplementary material:**

The online version of this article (10.1186/s12859-018-2549-8) contains supplementary material, which is available to authorized users.

## Background

Even after decades of research in the field of human genes, gene products and functions, understanding of genotype-phenotype relationship is far from complete. Biomolecules (genes, RNA, proteins, metabolites) interact with each other and environmental factors in order to accomplish various biological processes. Representing these interactions as biological networks (metabolic, protein-protein interactions, gene regulatory, co-expression) and their analyses provide insights in finding genes associated with cellular processes such as immune response, signalling pathways or with a complex disease like cancer [1].

**Fig. 1:**
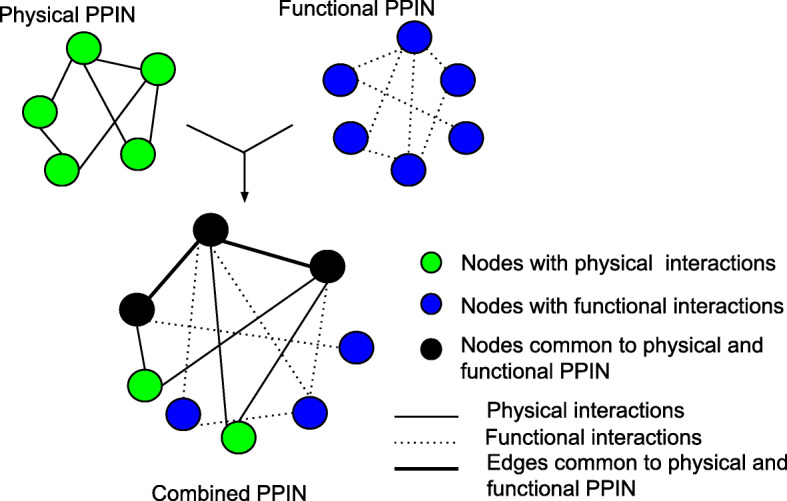
Illustrations of physical, functional and combined protein-protein interaction networks (PPIN)

Currently, 20,231 proteins of the human proteome have been identified [2] but the landscape of their interactions is only partially known. Protein interactions may be physical when their amino acid residues physically interact through electrostatic forces like hydrophobic or functional interactions when a protein influences the activity of another protein through regulation, co-expression, or some other genetic interaction [3, 4] (Fig. 1). Large scale experiments like yeast two-hybrid and affinity purification coupled to mass spectrometry identify physical protein interactions [5, 6] while high throughput expression techniques like microarray and RNA-seq elucidate functional links between proteins [7, 8].

Protein-protein interaction networks (PPIN) like most biological networks are believed to be modular in nature [4, 9, 10] and detecting functional modules of PPIN are vital for understanding gene-function associations and designing therapeutics. Topological modules are sub-networks where nodes within a module have dense connections as compared to the nodes of the other modules [11]. Functional module, on the other hand, is a sub-network that contribute to similar biological functions [4, 9]. Computational methods accurately inferring functional and disease modules of the human proteome would be of paramount importance for studying cellular and disease mechanisms.

Numerous computational algorithms have been attempted on biological networks in order to identify modules by using networks’ topological properties based on node neighbours [12], edge weights [13] and modularity [14, 15]. Other sub-network identifying algorithms including those finding core and loop structures [16, 17], cliques [18] and frequent graph patterns [19] have also been attempted to find topological modules in biological networks. However, only a few studies have compared their functional properties and their relevance to functional modules [20–22]. Usual approach to evaluate the functional significance of topological modules is to perform functional enrichment analysis and decide on the significantly enriched biological functions [21, 23, 24]. This approach is however inconclusive of determining functional coherence and specificity of topological modules [25]. In present work, we introduce a novel *functional specificity* measure that encompasses both functional homogeneity and heterogeneity of the topological modules. Top ranked topological modules are thereby identified and validated for their functional specificity.

We combine functional interactions inferred from expression data [26, 27] and physical interactions of PPIN [6, 16] to provide holistic functional attributes to protein nodes and interactions of the network for the determination of functional modules [28–30]. Though several studies have reported characteristics of resulting modules of different biological networks [13, 17, 21], there is a need of a systematic study elucidating the effects of using both functional and physical interactions of PPIN on detecting topological and functional modules. Previously, Theofilatos et al. and Lubovac et al. have applied weighted PPIN to predict protein complexes using a Markov clustering based approach and ranking measure on the basis of weighted neighborhood property, respectively [31, 32]. But here we investigate the role of edge weights incorporated from gene functional similarities in the modular detection of PPIN.

Our contributions in this study are (i) evaluation of functional coherence and specificity of the topological modules of the human proteome by using novel measures, (ii) determination of the effect of using both direct physical and indirect functional links of PPIN on detection of functional modules, and (iii) systematic analysis of incorporating functional context of interactions as edge weights using functional similarities of genes. We have used three different PPIN datasets of the human proteome and Louvain community detection algorithm [14] for modular detection. The weighted PPIN were generated by calculating functional similarity between interacting proteins by using molecular functions, biological processes and cellular components of Gene Ontology (GO) [33]. We also elaborate on how physical and functional interactions between proteins affect functional diversity of topological modules.

## Results

### Physical and functional PPIN

The present study considers three types of human PPIN based on physical, functional, and combined interactions as given in Table 1. The strengths or weights of protein-protein interactions with respect to their functional context (MF, BP and CC) are calculated from functional similarities of respective GO context, using Wang measure [34]. This led to nine sets of weighted PPIN and their network properties are listed in Table 2.

**Table 1:** Properties of different binary PPIN: physical (P), functional (F), and combined (C)

Network	Nodes	Edges	Avg. degree	Avg. path length	Diameter	Edge density	Clustering coeff.	Giant component size
P	13,269	98,013	14.73	6.95	11	0.0011	0.15	13,177
F	11,362	613,865	108.06	3.14	11	0.0095	0.26	11,271
C	15,562	700,640	90.04	6.10	11	0.0057	0.20	15,518

**Table 2:** Properties of weighted PPIN: physical (P), functional (F) and combined (C) PPIN weighted by functional contexts: molecular function (MF), biological process (BF) or cellular components (CC)

Network	Nodes	Edges	Avg. degree	Avg. path length	Diameter	Edge density	Clustering coeff.
P-MF	13,269	98,013	9.06	1.54	5.73	0.0007	0.133
P-BP			5.25	0.60	5.13	0.0004	0.136
P-CC			8.85	1.40	5.36	0.0007	0.135
F-MF	11,362	613,865	43.96	0.42	4.77	0.0038	0.223
F-BP			26.12	0.27	5.06	0.0023	0.222
F-CC			46.90	0.64	5.45	0.0040	0.226
C-MF	15,562	700,640	38.82	0.63	5.03	0.0025	0.176
C-BP			22.76	0.28	4.12	0.0015	0.178
C-CC			40.53	0.66	4.36	0.0026	0.180

PPIN like other biological networks such as metabolic and gene-regulatory networks are characterised by specific interactions between proteins (nodes) and functions of proteins and therefore demonstrate small world properties (i.e., short path length) and scale free characteristics (i.e., few nodes with large number of neighbours) (Tables 1 and 2).

### Topological modules

Topological modules of binary and weighted PPIN were detected using Louvain algorithm and analysed to investigate how (i) different interactions (physical and functional) and (ii) different biological contexts (i.e., MF, BP and CC ontologies) affect the functional properties of the modules.

**Fig. 2:**
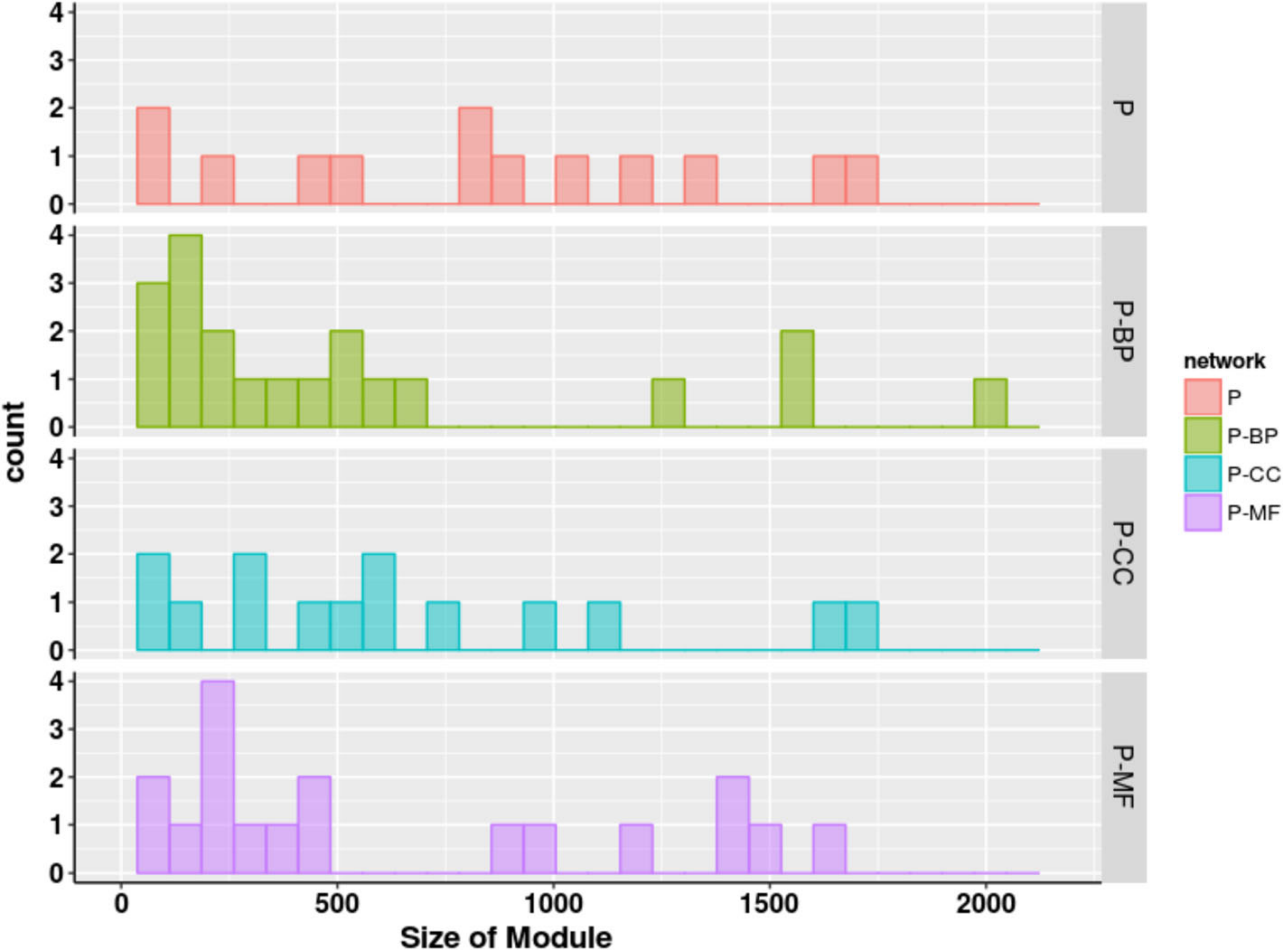
Size distributions for modules detected using Louvain algorithm in physical networks of human proteome: x-axis represents the size of modules while y-axis represents the count of meso-modules of size more than 10 nodes. P denotes the binary physical network while P-MF, P-BP and P-CC denote the weighted networks with edges scored according to functional similarity based on molecular functions (MF), biological process (BP) and cellular component (CC), respectively

**Fig. 3:**
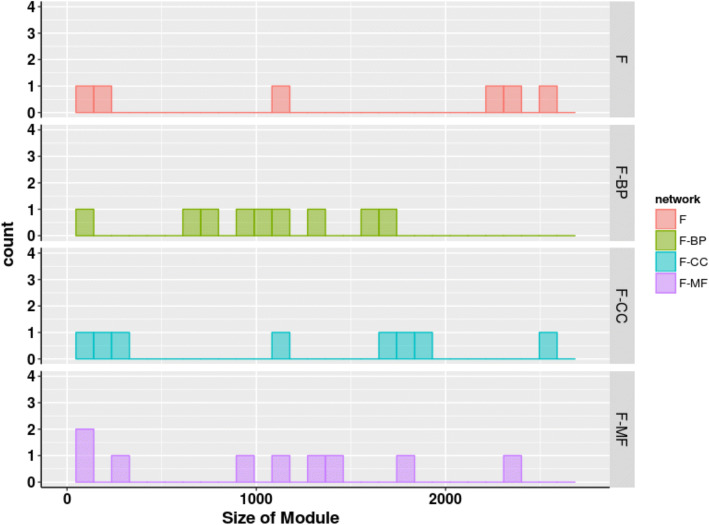
Size distributions of modules detected using Louvain algorithm in functional networks of human proteome. x-axis represents the size of modules while y-axis represents the count of meso-modules of size more than 10 nodes. F denotes the binary functional network while F-MF, F-BP and F-CC denote the weighted networks with edges scored according to similarity based on molecular functions (MF), biological process (BP), and cellular component (CC), respectively

**Fig. 4:**
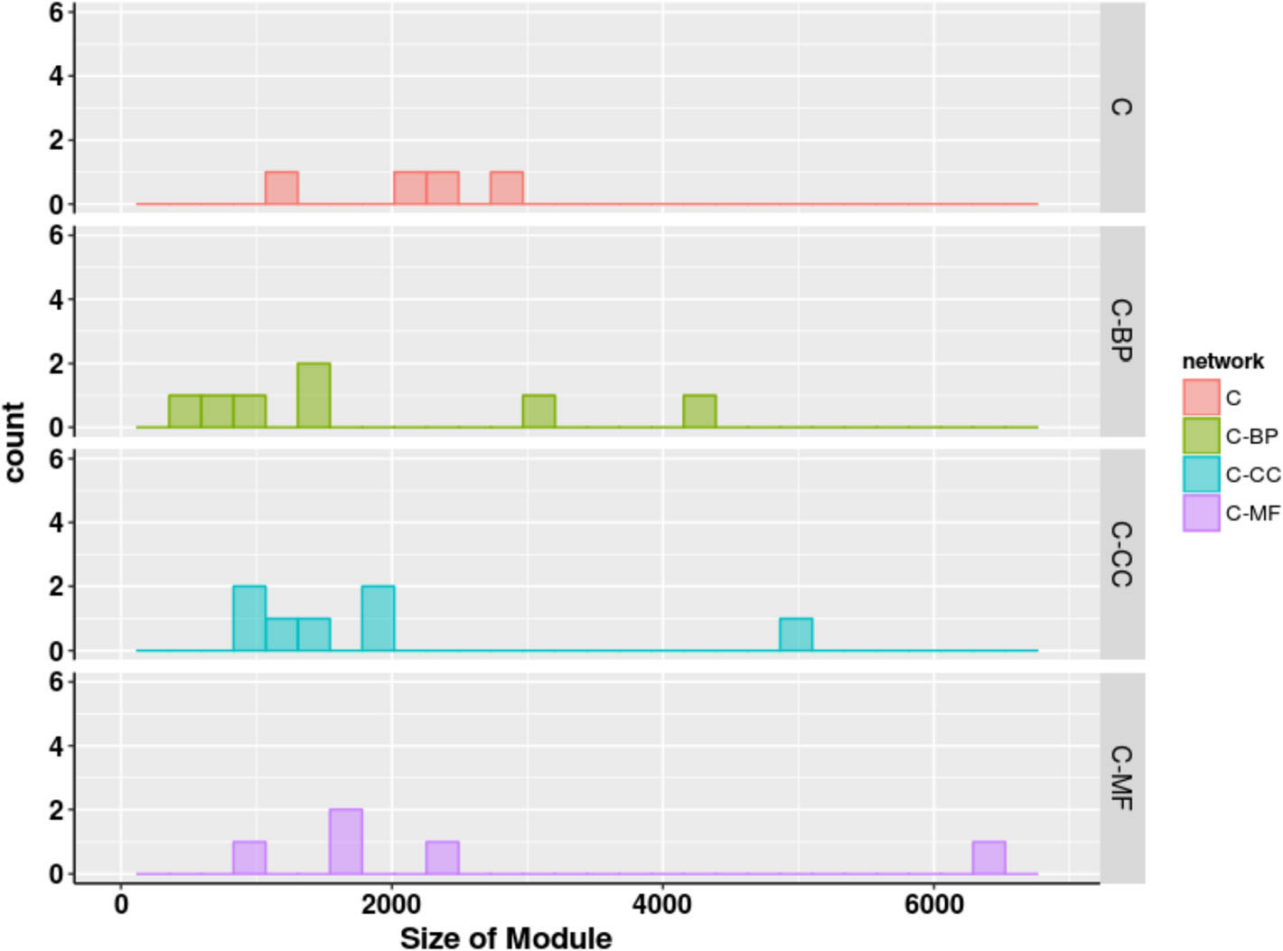
Size distributions for modules detected using Louvain algorithm in combined networks (physical and functional) of human proteome. x-axis represents the size of modules while y-axis represents the count of meso-modules of size more than 10 nodes. C denotes the combined physical network while C-MF, C-BP and C-CC denote the weighted networks where edges scored according to similarity based on molecular functions, biological process (BP), and cellular components, respectively

As shown in Table 3, the number of modules predicted for different networks vary considerably although the modularity values remain almost the same. We note that the number of modules predicted for weighted networks (1586 to 2912) is much more than that of binary networks (34 to 64), but only 0.3 to 1.2% of these modules are mesoscale (size> 10) as compared to 20–27% of binary networks. A closer inspection of Figs. 2, 3 and 4 finds that most of the modules are of size two, corresponding to isolated protein pairs whose interactions with others is yet be known or weak.

**Table 3:** Properties of topological modules of different PPIN

Network	Modularity	Number of Modules	Mesoscale^a^ modules (%)	Largest Module Size	Network edge density	Module edge density
P	0.43	64	26.6	2150	0.0011	0.0014 ± 0.01
P-MF	0.46	1586	1.2	1658	0.0007	0.0006 ± 0.0003
P-BP	0.53	2213	1.0	1988	0.0004	0.0004 ± 0.0002
P-CC	0.45	1754	0.9	2159	0.0007	0.0006 ± 0.0003
F	0.52	54	20.4	2730	0.0095	0.007 ± 0.004
F-MF	0.52	1777	0.6	2367	0.0038	0.005 ± 0.003
F-BP	0.55	1999	0.7	1998	0.0023	0.002 ± 0.001
F-CC	0.50	1700	0.7	2565	0.0040	0.009 ± 0.015
C	0.51	34	23.5	6882	0.0057	0.004 ± 0.003
C-MF	0.50	2391	0.3	6484	0.0025	0.003 ± 0.003
C-BP	0.53	2912	0.4	4186	0.0015	0.002 ± 0.001
C-CC	0.48	2430	0.5	4869	0.0026	0.002 ± 0.001

^a^Mesoscale modules refer to the modules with size more than 10

### Biological relevance of PPIN modules

**Fig. 5:**
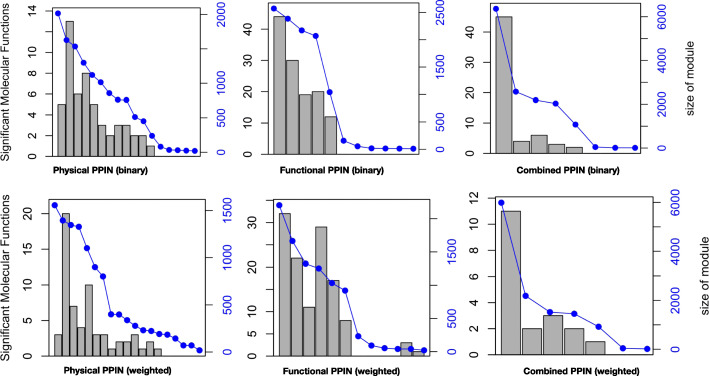
Functional enrichment analyses of topological modules: (a) and (b) show distributions of enriched molecular functions in topological modules of PPIN networks. X-axis, Y-axis (left) and Y-axis (right) represent the modules, number of statistically significant GO terms, and size of modules, respectively. See Additional file 1: Figure S2 for the set of enriched biological processes and cellular locations in the modules

More importantly, proteins in topological modules ought to share the same functional profile**.** To study functional relevance of topological modules in the human proteome, mesoscale modules from all networks were tested for their biological relevance by using functional enrichment analysis. The enriched function set *F* is given by the union of all significantly enriched functions across topological modules and functional specificities of the set of enriched functions were computed for each PPIN. Figure 5 (and Additional file 1: Figure S2) shows the distribution of significantly enriched biological functions and size of topological modules of binary and weighted physical PPIN.

### Functional homogeneity and specificity of topological modules

Functional homogeneity of a module quantifies functional consistency of a topological module as defined by the maximal fraction of proteins associated with a biological function. The homogeneity ranges from 0 to 1 where a value of 1 indicates that all genes in the module exhibit that function. A module’s heterogeneity value estimates how specific a function is for a particular module.

A recent study of human proteome [35] discussed how most of the topological modules are functionally diverse despite high homogeneity values. In our study, we further this observation by including functional interactions and incorporating the weights to PPIN. As shown in Table 4, the MF and BP homogeneity values are observed to be higher (0.79 and 0.59) for physical networks than functional networks (0.64 and 0.57) whereas cellular localizations (~ 0.7) do not vary much across different networks. We conclude that functional interactions lead to low homogeneity values in networks because they mostly represent cross talks between modules with not much variations in cellular localizations. For example, cross talks in TGF-beta signalling is known to be involved in many developmental defects and cancer [36]. This observation concurs with homogeneity values derived in gene-disease associations (a type of functional interactions) in disease networks [24, 37].

**Table 4:** Functional homogeneity of mesoscale modules detected by Louvain algorithm, evaluated using three ontologies: MF, BP, and CC

PPIN		MF			BP			CC		
		max	mean	std	max	mean	std	max	mean	std
Physical	Binary	0.81	0.79	0.01	0.85	0.59	0.24	0.78	0.75	0.04
	Weighted	0.83	0.80	0.02	0.75	0.42	0.31	0.80	0.77	0.01
Functional	Binary	0.71	0.64	0.12	0.72	0.57	0.25	0.8	0.72	0.25
	Weighted	0.71	0.42	0.22	0.74	0.60	0.25	0.8	0.76	0.07
Combined	Binary	0.74	0.73	0.01	0.70	0.58	0.25	0.77	0.75	0.01
	Weighted	0.73	0.72	0.002	0.72	0.45	0.29	0.76	0.75	0.01

Table 5 shows heterogeneity values for enriched functions of the modules. On average, molecular function homogeneity was observed to be higher than bioprocess homogeneity for physical (0.80 > 0.42) and combined (0.72 > 0.45) networks except for functional networks (0.42 < 0.60). But homogeneity and heterogeneity values are more varied (high standard deviation) for functional PPIN than physical and combined. Thus, it is advantageous to integrate physical protein interactions with expression based networks for functional analyses as attempted in some reported studies [29, 38].

**Table 5:** Functional heterogeneity of modules detected by Louvain algorithm, calculated for all the enriched functions

PPIN		MF			BP			CC		
		min	mean	std	min	mean	std	min	mean	std
Physical	Binary	0.05	0.07	0.05	0.04	0.09	0.06	0.05	0.09	0.08
	Weighted	0.04	0.08	0.12	0.04	0.09	0.05	0.05	0.21	0.20
Functional	Binary	0.09	0.22	0.14	0.09	0.17	0.12	0.09	0.25	0.16
	Weighted	0.08	0.20	0.14	0.07	0.16	0.15	0.09	0.24	0.16
Combined	Binary	0.14	0.20	0.12	0.14	0.25	0.15	0.14	0.28	0.21
	Weighted	0.13	0.21	0.18	0.07	0.10	0.05	0.09	0.31	0.19

### Effect of the resolution limit on module detection in PPIN

Modularity-based algorithms for module detection often suffer from resolution limit [39] as the scale of modularization depends upon the inter-connectedness of the modules. This leads to the inability to detect smaller modules in a given network. To study the effect of resolution limit in detecting topological modules, we also implemented the Incremental Louvain algorithm [35], which first finds modules by maximizing modularity while incrementally modularizing larger modules into smaller sub-networks, thus converging the algorithm for modules with size greater than a threshold size.

Here, we observed on average eight times more mesoscale modules as compared to the Louvain algorithm, the majority of modules being smaller in the size range of 10 to 200 (Additional file 1: Figure S4). In case of smaller modules detected using Incremental Louvain algorithm, an increase in the homogeneity values is observed when indirect functional interactions are combined with physical PPI (Tables 6 and 7). While functional homogeneities of modules detected with the Louvain algorithm decreased when functional interactions are introduced into PPI network. This phenomenon can be simply attributed to difference in module sizes. When compared with respect to three ontologies, the homogeneity of modules shows on average 3.4% decrease for MF, 47.08% increase for BP and 4.6% decrease in CC. And heterogeneity values showed large percentage of decrease for these smaller modules (85.1, 78.9 and 87% decrease in MF, BP and CC, respectively). Weighting interactions in PPI network improves homogeneity of these modules but no change in heterogeneity values is observed.

**Table 6:** Functional homogeneity of mesoscale modules detected by Incremental Louvain algorithm, evaluated using three ontologies: MF, BP, and CC

PPIN		MF			BP			CC		
		max	mean	std	max	mean	std	max	mean	std
Physical	Binary	1	0.57	0.28	1	0.67	0.29	1	0.65	0.28
	Weighted	1	0.63	0.27	1	0.81	0.20	1	0.76	0.22
Functional	Binary	1	0.59	0.25	0.97	0.73	0.22	1	0.72	0.23
	Weighted	0.97	0.69	0.25	1	0.85	0.16	1	0.72	0.24
Combined	Binary	1	0.60	0.26	1	0.72	0.26	1	0.70	0.24
	Weighted	0.98	0.65	0.26	1	0.82	0.19	1	0.74	0.21

**Table 7:** Functional heterogeneity of the modules detected by Incremental Louvain algorithm, calculated for all the enriched functions

PPIN		MF			BP			CC		
		min	mean	std	min	mean	std	min	mean	std
Physical	Binary	0.008	0.017	0.02	0.008	0.02	0.03	0.008	0.02	0.03
	Weighted	0.007	0.017	0.02	0.008	0.03	0.04	0.008	0.03	0.04
Functional	Binary	0.01	0.02	0.03	0.01	0.03	0.03	0.01	0.03	0.04
	Weighted	0.01	0.03	0.03	0.01	0.04	0.04	0.01	0.03	0.05
Combined	Binary	0.007	0.02	0.02	0.007	0.02	0.02	0.007	0.02	0.04
	Weighted	0.008	0.02	0.02	0.007	0.02	0.03	0.01	0.03	0.05

### Functionally specific modules

**Fig. 6:**
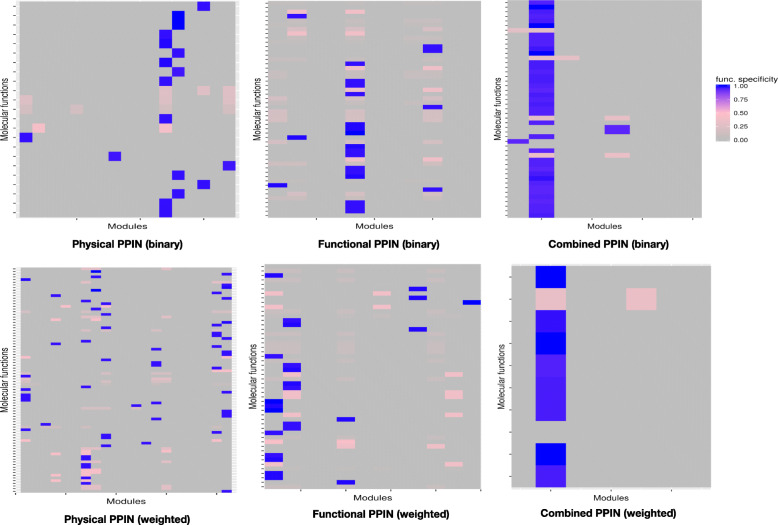
Functional specificity of significantly enriched molecular functions of topological modules. See Additional file 1: Figure S3 for specificity scores of topological modules for BP and CC enrichment

The specificity of a particular function takes both its homogeneity within the module and its diversity across the modules into account. The normalized specificity scores for all significantly enriched functions across modules are summarized in Fig. 6. As seen from the patterns of homogeneity and heterogeneity values, physical PPIN produce more functionally specific modules (highly homogenous and less diverse) than functional and combined PPIN, underscoring the benefit of including proteomics while analysing expression based networks in the identification of functional modules.

Topological modules were ranked using the specificity score and we labelled the modules with normalized specificity greater than 0.90 as functionally *specific modules* and the others as *general modules*. Table 8 summarizes the biological functions and Table 9 enlists enriched biological pathways of specific modules. Main functions specific to the modules were enzymatic activities like kinase, hydrolase, and transferase; and protein and nucleotide binding activities. About 36 to 55% of topological modules in binary and 14 to 32% of in weighted networks were classified as specific modules according to above mentioned criteria. More number of modules are found to be functionally specific (55%) in physical PPIN as compared to functional and combined PPIN (Table 8). This is in agreement with the effect of heterogeneity and homogeneity values of physical networks. This maybe imparted to the fact that direct interaction between proteins which are elucidated through high throughput screening experiments [6, 40] are more often studied and more popularly annotated with functions and that gene-function associations as annotation based functional enrichment analysis are affected by missing annotations. See Additional file 1: Tables S1 and S2 for specific modules enriched in biological processes and cellular locations.

**Table 8:** The percentage (%), mean size, and summary of molecular functions of the specific modules of physical (P), functional (F) and combined (C) PPIN

Network	%	Mean Size (std.)	Specific molecular functions of modules
P	0.55	1523 (390)	Module1: cytoskeletal protein binding; Module2: receptor activity; Module3: cation binding, dimerization/transcription factor activity; Module4: cyclic compound/RNA/chromatin binding, Wnt-activated receptor activity; Module5: DNA binding; Module6: pyruvate dehydrogenase (acetyl-transferring) kinase activity
P-weighted	0.32	1215 (262)	Module1: deacytylase activity; Module2: cyclic compound/nucleotide/ATP/nucleoside binding, kinase/transferase activity; Module3: amide/peptide binding; Module4: transferase activity; Module5: protein domain specific binding; Module6: DNA binding
F	0.36	2298(223)	Module1: RNA binding; Module2:ssDNA/ nucleotide/nucleoside/GTP/Mg ion binding binding, oxidoreductase/transferase/kinase/ activity, transmembrane transporter activity; Module3: ATPase/DNA helicase activity, chromatin binding
F-weighted	0.42	1036 (978)	Module1: hydrolase/transferase activity, TF/transcription regulator/transcription coactivator/transcription cofactor/transmembrane transporter activity, CCR5 chemokine receptor binding; Module2: ATPase/hydrolase activity, transmembrane transporter activity; Module3: kinase activity, DNA binding; Module3: ATPase activity; Module4: alcohol binding
C	0.38	4011 (2495)	Module1: lamin binding; Module2: cyclic compound/ion/DNA/enzyme/small molecule/ATP/chromatin/protein kinase/nucleotide/nucleoside binding, transcription regulator/protein dimerization/kinase/nucleoside-triphosphatase/phosphotransferase activity; Module3: macrolide/FK506 binding
C-weighted	0.14	6484(0)	Module1: ion/cyclic compound/DNA/cation/enzyme/identical protein binding, catalytic/transferase activity

**Table 9:** The top enriched protein pathways in the specific modules of physical (P), functional (F) and combined (C) PPIN. Pathways are mapped using PANTHER Pathway database (http://www.pantherdb.org/pathway/)

Network	Specific pathways of modules
P	Inflammation mediated by chemokine and cytokine signalling pathway; gonadotropin-releasing hormone receptor pathway; Wnt signalling pathway; Integrin signalling pathway; CCKR signalling map; Heterotrimeric G-protein signalling pathway; Angiogenesis; PDGF signalling pathway; Apoptosis signalling pathway; EGF receptor signalling pathway
P-weighted	Inflammation mediated by chemokine and cytokine signalling pathway; gonadotropin-releasing hormone receptor pathway; Wnt signalling pathway; Integrin signalling pathway; PDGF signalling pathway; Heterotrimeric G-protein signalling pathway; Angiogenesis; Apoptosis signalling pathway; CCKR signalling map; Angiogenesis; EGF receptor signalling pathway; FGF signalling pathway; Huntington disease; Cadherin signalling pathway; Alzheimer disease-presenilin pathway
F	Inflammation mediated by chemokine and cytokine signalling pathway; gonadotropin-releasing hormone receptor pathway; Wnt signalling pathway; Integrin signalling pathway, CCKR signalling map; Angiogenesis; EGF receptor signalling pathway; Huntington disease; Alzheimer disease-presenilin pathway; TGF-beta signalling pathway; PDGF signalling pathway; Heterotrimeric G-protein signalling pathway; Nicotinic acetylcholine receptor signalling pathway
F-weighted	Inflammation mediated by chemokine and cytokine signalling pathway; gonadotropin-releasing hormone receptor pathway; Wnt signalling pathway; Integrin signalling pathway, CCKR signalling map; Angiogenesis; EGF receptor signalling pathway; FGF signalling pathway; Heterotrimeric G-protein signalling pathway; PDGF signalling pathway; Huntington disease; Alzheimer disease-presenilin pathway; B-cell activation; Parkinson disease; Insulin/IGF pathway; Interleukin signalling pathway; Ionotropic glutamate receptor pathway; Mannose metabolism; Pyridoxal-5-phosphate biosynthesis; PDGF signalling pathway
C	Inflammation mediated by chemokine and cytokine signalling pathway; gonadotropin-releasing hormone receptor pathway; Wnt signalling pathway; Integrin signalling pathway, CCKR signalling map; Angiogenesis; Heterotrimeric G-protein signalling pathway; EGF receptor signalling pathway; PDGF signalling pathway; Huntington disease; FGF signalling pathway; Apoptosis signalling pathway
C-weighted	Inflammation mediated by chemokine and cytokine signalling pathway; gonadotropin-releasing hormone receptor pathway; Wnt signalling pathway; Integrin signalling pathway, CCKR signalling map; Angiogenesis; Heterotrimeric G-protein signalling pathway; EGF receptor signalling pathway; FGF signalling pathway; Cadherin signalling pathway

### Biological validation by pathway enrichment analysis

To validate biological relevance of top ranked specific modules, their enrichment with genes from experimentally known biological pathways was computed. Four gene sets of known pathways were considered: glycolysis, transcriptional regulation, lung cancer and breast cancer, and their details [40–42] are given in Table 10. Breast and lung cancer pathway set has a total 363 and 300 genes, out of which 347 and 286 are present in the physical, 260 and 219 in the functional and 349 and 288 in the combined PPIN. Out of 244 genes from glycolysis pathway, 158 are present in the physical, 187 in the functional and 226 in the combined PPIN.

**Table 10:** Details of biological pathways used for validation of functional modules

Biological Pathway	No. of genes	Overlap with			Source
		Physical PPIN	Functional PPIN	Combined PPIN	
Glycolysis	262	203	187	229	KEGG [41], MSigDB [42]
Transcriptional regulation	1705	1554	1243	1640	Rolland et al. [40]
Lung cancer	300	286	219	288	Rolland et al. [40]
Breast cancer	363	347	260	349	Rolland et al. [40]

**Fig. 7:**
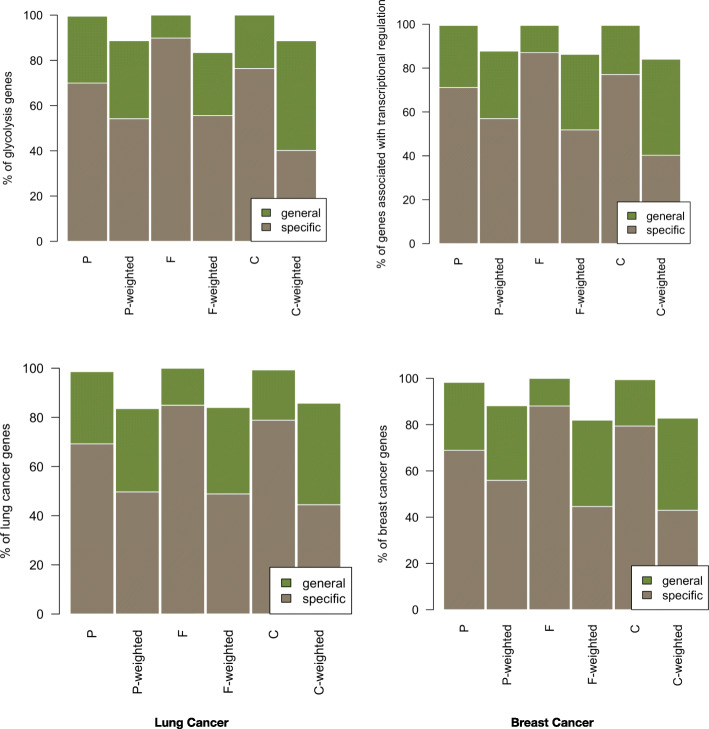
Overlap of specific topological modules (specificity score > 0.9) and general topological modules (specificity score < 0.9) with experimentally known biological pathways: glycolysis, transcriptional regulation, lung cancer and breast cancer. Bars represent overlap of genes involved in biologically validated pathways with specific modules (brown colour) and general modules (green colour). Topological modules are detected via molecular function enrichment for binary and weighted physical(P), functional(F) and combined (C) PPINs

The overlapped fractions of genes of known pathways to those in specific and general modules were calculated in order to estimate validity of the topological modules. As shown in Fig. 7, specific modules from binary combined PPIN retrieved ~ 79% of breast and lung cancer genes as compared to 43–45% by modules of weighted PPIN. In a similar fashion, for physical and functional PPIN, specific modules of binary PPIN were enriched with more cancer pathway genes (69 and 89% for breast cancer, 69 and 85% for lung cancer) than respective modules from weighted PPIN (56 and 45% for breast cancer, ~ 49% for lung cancer). Specific modules of binary networks were also highly enriched with 70, 90, and 76% of glycolysis genes and 71, 87 and 77% of transcriptional regulation genes in physical, functional and combined networks, respectively.

## Discussion

The three different PPIN (physical, functional and combined) were modularized and their functional relevance was analysed using functional enrichment analysis. As observed from Table 1, physical PPIN are sparser (have high average path length and low edge density) than functional PPIN, resulting due to high number of functional interactions and noise in the gene expression experiments. For weighted networks (Table 2), the edges with low functional similarity between proteins reduce the average path length to lower values than binary PPIN (ranges from 0.2 to 1.5 as compared to 3.1 to 6.9). There is a high overlap between functional and physical PPIN with 9069 common nodes between the two, underlining that most physical interactions also exert functional interactions. However, small amount of non-overlapping edges between physical and functional PPIN suffices to cause changes in edge density and clustering coefficient for the combined network.

When modularized using Louvain algorithm, size distribution of topological modules in three PPIN (Figs. 2, 3, 4 and Table 3) shows that weighting interactions with functional similarities of proteins removes weak protein-protein interactions in PPIN and leads to higher number of compact modules.

Figure 5 (and Additional file 1: Figure S2) shows the functionally enriched GO terms in the PPIN modules. The number of enriched cellular functions and processes are observed to be higher for the weighted PPIN despite the smaller size of the modules. The number of cellular locations decreases however with the inclusion of weights of protein interactions. Overall, combined PPIN are enriched by more GO terms, with biological processes approximately 1.5 to 3 times more, molecular functions up to 3 times more and cellular locations approximately 1.4 times more, than those in physical and functional binary PPIN.

Functional homogeneity analysis (Tables 4 and 5) shows Physical PPIN modules to be more specific than functional networks, in case of molecular functions as compared to bioprocesses and cellular localizations. Overall, homogeneity and heterogeneity values are not much different when weighted interactions are considered, indicating that topological modules are more resilient to edge weights when functional annotations are considered. We also conclude that topological modules in PPIN are more homogeneous and specific in molecular functions, and less homogeneous (diverse) in terms of biological processes. This is in agreement with the fact that a biological process may involve multiple sets of molecular functions and thus functional modules map to a number of molecular functions but less number of biological processes. Most importantly, the results indicate that the functional modules are observed to be more homogenous and specific when direct interactions in PPIN are also considered (as seen in the combined network), a fact to kept in mind when identifying biologically relevant modules by using computational methods. To study the effect of resolution limit on functional properties of modules, three PPIN were modularized using Incremental Louvain Algorithm that resulted in modules, eight times more in number but smaller in size than Louvain (Additional file 1: Figure S4). Despite the differences, enrichment analyses of modules from both type of algorithms show that physical networks are more specific than functional ones (see Tables 6 and 7). Thus topological modules are more specific and homogeneous when direct interactions are considered with indirect functional associations (such as derived from co-expression or microarray based experiments).

A specificity score is introduced in this study that considers both functional homogeneity and heterogeneity of a module. Topological modules with specificity score greater than 0.90 were labelled functionally *specific modules* and the others as *general modules*. Table 8 shows that physical PPIN modules are more enriched in specific modules than functional PPIN. As seen in Fig. 6 and Additional file 1: Figure S3, the modules appear to become smaller and the biological functions re-distributed into more number of highly specific modules when edge weights are introduced to physical and functional protein interaction networks. However, combining functional interactions with physical interactions led to formation of few and larger specific modules. This limitation due to increasing module size can be handled by optimizing modularizing algorithm for detecting smaller modules of high functional specificity in future and is beyond the scope of present study.

Biological relevance of top ranked specific modules in physical, functional and combined PPIN was evaluated on the basis of their enrichment with genes from experimentally known biological pathways such as glycolysis, transcriptional regulation, lung cancer and breast cancer. As shown in Fig. 7, specific modules are overall found to be more enriched than general modules for all four biological pathways, but the specific modules from binary PPIN were observed to be highly enriched than those of weighted PPIN. This indicates that the specific modules obtained by using specificity scores of enriched functions are highly enriched with known functional and disease pathways. However, inclusion of weights did not improve the enrichment of biological and disease pathways in physical and functional networks.

## Conclusions

We systematically analysed functional properties of topological modules in human proteome and investigated the effect of physical and functional interactions in PPIN on functional specificity of modules. We also studied the contribution of weighting edges with functional similarities on topological modules. A specificity score was introduced to identify more accurate biologically relevant and specific modules. Functionally homogeneity was earlier used to evaluate functional value of topological modules detected in biological networks [24, 25] but failed to consider the heterogeneity of functional modules of biological networks due to a protein or gene mapping to a number of cellular processes. Thus, a set of proteins (in a module) are involved in more than one function and also a biological function is mapped to more than one module. In order to handle this, functional specificity was introduced which considers both functional homogeneity within the module and functional heterogeneity across the modules. The function specificity helps in identifying functional modules or specific functions of topological modules and one may use our methods to confidently map specific functions to topological modules of PPIN.

The topological modules detected using physical, functional and combined PPIN are found to be homogeneous, highly specific, and enriched in a number of significant biological functions, processes and cellular localizations (Fig. 5 and Additional file 1: Figure S2). Though weighted edges do not affect the homogeneity and heterogeneity of the modules, incorporating functional similarities of edge do help in identifying compact and highly specific functional modules based on topological properties.

Functional or indirect interactions are generally noisy as they are determined using statistical inferences from gene expressions based experiments and vary on tissue and patient sample basis [40]. But functional interactions encompass the whole interaction profile of genes involved in a cellular function or a disease and thus important for systematic analysis and prediction of functional modules. Present study provides a first hand insight into the effect of these different type of protein-protein interactions on topological modules of human proteome. We conclude that instead of using only co-expression based networks in identifying functionally relevant topological modules, one should combine the accuracy of physical interactions with the larger coverage of interactome landscape by functional networks. Though our methodology provides an edge over usual methods (like homogeneity, GO enrichment) for functional validation of topological modules and helps in identifying specific functions of these modules, it does not identify core components of a biological pathway. One limitation of our study is that our methods do not handle the overlapping modules and consider overlapping properties of functional modules. It would be interesting to study overlapping sub-modules, core modules, and the hierarchical organization of functionally specific topological modules as future work of this study.

## Methods

### Datasets

In a cellular machinery, proteins function as enzymes, transcription factors, receptors or structural proteins, and interact with other biomolecules. Protein interactions are either *physical* (direct) or *functional* (indirect). For studying the role of these two types of interactions on detection of modules of PPIN, three datasets were used: Physical, Functional and Combined (see Fig. 1, Table 1). These three datasets were prepared from HPRD (Human Protein Reference Database) (version Release9) [43] and STRING database (version 10) [44]; and include experimental information from other well-known databases like BIND, DIP, GRID, HPRD, IntAct, MINT and PID (updated till 14 May 2017). All the proteins were mapped to their Entrez gene ids. Details of data pre-processing are provided in Additional file 1.

1. **Physical PPIN** enlists curated binary interactions of proteins, representing physical or direct interactions that are determined using in vivo (e.g. co-immunoprecipitation), in vitro (e.g. GST pull-down assays) or yeast two-hybrid experiments.
2. **Functional PPIN** represents functional interactions of proteins, i.e., these proteins may or may not physically interact but they do participate in a biological function by influencing each other genetically through co-regulation or co-expression, which are determined using experimental techniques like microarray expression data analysis or double mutant analysis.
3. **Combined PPIN** is the inclusive set of both the physical and functional networks mentioned above.

### Weights for protein-protein interactions

Weighted PPIN are obtained by assigning functional similarities between proteins as edge weights, considering different GO domains: molecular function (MF), biological process (BP) and cellular component (CC). We used popular Wang’s semantic similarity measure [34, 45] to evaluate the functional similarity between genes (i.e., weights of protein-protein interactions).

### Module detection

Functional modules of PPIN correspond to communities or sub-networks of proteins having specific and similar biological functions [4, 46]. We chose the Louvain algorithm modular detection algorithm to find topological modules of PPIN because it has demonstrated excellent performance and low computational complexity on benchmark networks [20] (Lancichinetti & Fortunato, 2009). The Louvain algorithm finds the community or modular structure by optimizing the modularity *Q* (the quality function) of the network:

1 $$ Q={\sum}_{ij}\left({e}_{ij}-{\left({a}_i\right)}^2\right) $$

where *e*_*ij*_ is fraction of edges between modules *i* and *j*, and *a*_*i*_ is the fraction of edges connected to the nodes in module *i*. The modular structure is found by maximizing the modularity in an iterative manner. All the nodes in the network are assigned to independent modules in the beginning and the algorithm progressively merges two communities that best increase the modularity of the resulting network structure. Merging of nodes and modules continues until there is no further increase in the modularity of the network.

### Functional enrichment analysis

The functional enrichment analysis was performed in order to find the GO terms in MF, BP, and CC contexts, which are significantly represented (enriched) by the proteins in the predicted topological modules. The functional enrichment analysis was implemented using R package BioStats [47]. The statistical significance of a GO term in a module was estimated by evaluating its overrepresentation using a hypergeometric test. A functionally enriched module signifies that the number of genes observed to be annotated with a function (i.e., the GO term) is more than the expected number of genes annotated to that function. The ‘expected value’ for a function is the number of genes having that specific function in the given module, with respect to the reference list (whole list of human genes).

### Functional homogeneity and specificity of topological modules

In this section, we introduce measures to quantify functional homogeneity and heterogeneity of topological modules of PPIN. First, functional enrichment analysis is performed on the modules to identify biological functions (GO terms) that are significantly enriched (*p*-value < 0.0001) in the modules and the functions are ranked according to their significance values. Systematic estimation of *p*-value is done using a set of detailed experiments explained in Additional file 1. We selected the enriched functions for each module and identified the set *F* of enriched functions in all the modules.

Homogeneity of a module with respect to a particular function is computed by the proportion of genes annotated by the function. That is, the homogeneity of a function *f* ∈ *F* within a module is given by

$$ homogeneity=\frac{n_f}{N} $$

where *n*_*f*_ is the number of genes annotated by the function and *N* is the total number of genes in the module. The *functional homogeneity* (*H*) of a module is defined as the homogeneity of maximally enriched function in the module. The *heterogeneity* of a function is defined as the proportion of the modules where the function *f* ∈ *F* is enriched. That is,

$$ heterogeneity=\frac{k_f}{K} $$

where *k*_*f*_ is the number of modules enriched with function *f* and *K* is the total number of modules detected in PPIN.

Functional homogeneity measures functional coherence of the modules while functional heterogeneity indicates how exclusive the modules are for the function across all predicted modules. To combine functional homogeneity and heterogeneity of a module, *functional specificity* for an enriched function is defined as follows:

2 $$ specificity= homogeneity+\frac{1}{heterogeneity} $$

The values of specificity scores across all enriched functions are normalized to a range between 0 and 1. The functional specificity value measures how exclusively the module is enriched by the specific biological function. Modules are ranked using the functional specificity score and the top ranked modules are considered as highly specific modules.

## Additional file

Additional file 1: Supplementary information. (PDF 1521 kb)

## Abbreviations

BP: Biological Process
C: Combined
CC: Cellular Component
F: Functional
GO: Gene ontology
MF: Molecular function
P: Physical
PPI: Protein-protein interactions
PPIN: Protein-protein interaction networks
