# Supplementary material for: Functional homogeneity and specificity of topological modules in human proteome
Source: BMC Bioinformatics. 2019 Feb 4;19(Suppl 13):553. doi: 10.1186/s12859-018-2549-8 (PMC7394330; doi:10.1186/s12859-018-2549-8)
Supplement: Supplementary file 1 — Supplementary information. (PDF 1521 kb) [file 12859_2018_2549_MOESM1_ESM.pdf]

Functional homogeneity and specificity of topological modules in human proteome

Supplementary Information

A. Network properties

A network is represented by a graph G (V,E) where V is the set of n nodes and E is the set of edges between these vertices.

Average Degree: The degree K_i of a vertex i is given by the number of edges connected to it. Average degree of the network is calculated by:

$$< K > = \frac{\sum_{i=1}^{n} K_i}{n}$$

Network Density: The density of a graph is given by the ratio of number of edges E in the graph and the number of possible edges $\binom{n}{2}$ between n nodes.

Average path length: It represents the average distance required to travel from one node to the other through connected edges. It is calculated by adding the shortest paths between all possible pairs of nodes and dividing by the total number of possible pairs.

Diameter: The diameter of the graph is the length of the longest geodesic (longest of the all calculated shortest paths) in a network.

Clustering coefficient: Clustering coefficient (C_i) or transitivity of a node is the probability of how the adjacent neighbours of a node are connected. It is given by the ratio of existing edges between a node's neighbours and the possible edges between them. The clustering coefficient of a network is the average of the clustering coefficients of all nodes in the network.

$$C_i = \frac{2e_i}{k_i(k_i - 1)}$$

where k_i is the number of neighbours of node i and e_i is the number of existing edges between them.

B. Preprocessing PPIN datasets from public databases

Physical PPIN: HPRD is a manually curated database containing reliable protein-protein interactions. STRING database combines various experiments (experiments, co-expression, co-occurrence etc) and gives scores for interactions, which represents the source and strength of the evidence of interaction. For the present study, we included all the interactions from HPRD. The interactions only having action ‘binding’ from species ‘*Homo sapiens*’ from STRING were included in the analysis. Further, physical PPIN from STRING includes interactions with experimental evidence, i.e., experiments score >0

Functional PPIN: Functional interactions were derived from STRING, with interactions with experimental evidence, i.e., coexpression_transferred score >0). Since it is important to have PPI inclusive of all (weak and strong) experimental evidences for the purpose of this study, we have selected all the interactions with non-zero scores mentioned in functional and physical interactions.

C. Systematic estimation of p-value for GO enrichment analysis

The statistical significance or p-value refers to the probability that given a statistical model, an event is statistically significant as compared to random events. But a p-value alone does not measures the probability that a hypothesis is true nor it measures the size of effect or the important of a result [1, 2].

The optimal p-value to decide on statistical significance for enriched functions in the modules was determined by considering the odds-ratio (odds of being GO term X | in significant set/odds of being GO term X |not in significant set). Experiments with different p-values: 0.05, 0.01, 0.005, 0.001, .0005, .0001 were performed. For enriched GO terms in all modules of three PPIN, odds-ratios were calculated and compared for different p-values (suppl. Figure S5). The p-value = 10^{-4} gave the maximum modularity with highest odds ratio of the GO terms and therefore selected for the analysis.

Results and Discussion:

Optimum p-value was selected as the p-value that gave the highest odds ratio of the GO terms. Effect size denotes the difference between the number of enriched terms at diffferent p-values. The optimum p-value with maximum number of modules having GO terms with high odd ratio for all three networks was found to be 10^{-4} (Table SC.1). The effect size for terms at p-values 10^{-3} & 10^{-4} was also small as compared to that of 10^{-2} & 10^{-3}. Thus p-value of 10^{-4} was selected as the optimum value without losing significant number of enriched GO terms.

In order to validate our choice of p-value, we calculated enriched functions of topological modules at a higher p-value of 10^{-3} and analysed the change in homogeniety and heterogeneity values at p-value = 10^{-4} (Tables 4 & 5; Tables S3 & S4). Though there is a slight decrease in homogeneity and heterogeneity of modules at different p-values, the effects of direct and functional interactions on homogeniety and heterogeneity values were not affected by the choice of p-value.

Table SC.1 Statistical significance test results at different p-values. Optimum p-value denotes the p-value that gave highest odds ratio of the GO terms. Effect size denotes the difference between the number of enriched terms at diffferent p-values (10^{-2},10^{-3}) and (10^{-3},10^{-4}).

**PPI**	**GO**	**Optimum p-value**	***EFF SIZE(10^{-2},10^{-3})**	***EFF SIZE(10^{-3},10^{-4})**
P	MF	0.0001	3.98	0.53
	BP	0.0001	1.74	0.48
	CC	0.0001	1.17	0.38
F	MF	0.0001	1.34	0.38
	BP	0.0001	2.03	0.42
	CC	0.0001	0.75	0.22
C	MF	0.0001	1.52	0.31
	BP	0.0001	1.00	0.23
	CC	0.0001	0.79	0.22

*EFF SIZE(p1,p2)= |mean (Tp1) -mean (Tp2)|/sd(Tp1,Tp2) where Tp1 and Tp2 are no. of significant terms at p-value p1 and p2

References:

[1] Wasserstein, Ronald L., and Nicole A. Lazar. "The ASA's statement on p-values: context, process, and purpose." *The American Statistician* 70.2 (2016): 129-133.

[2] Benjamin, Daniel J., et al. "Redefine statistical significance." *Nature Human Behaviour* 2.1 (2018): 6.

D. Supplementary Tables

Table S1. Fraction of specific modules enriched in biological processes. S_k denotes the fraction of specific modules in the network.

**Network**	**S_k**
P	0.70
P-weighted	0.61
F	0.64
F-weighted	0.69
C	0.5
C-weighted	0.46

Table S2. Fraction of specific modules enriched in cellular locations. S_k denotes the fraction of specific modules in the network.

**Network**	**S_k**
P	0.29
P-weigted	0.38
F	0.36
F-weighted	0.36
C	0.37
C-weighted	0.27

Table S3. Functional homogeneity of mesoscale modules of PPIN from Louvain algorithm, evaluated using three ontologies: MF, BP, and CC. p-value = 10^{-3}

**PPIN**		**MF**			**BP**			**CC**		
		max	mean	std	max	mean	std	max	mean	std
Physical	Binary	0.78	**0.71**	0.18	0.86	0.64	0.25	0.75	0.71	0.11
	Weighted	0.8	**0.72**	0.16	0.84	0.48	0.28	0.78	0.70	0.16
Functional	Binary	0.68	0.35	0.23	0.72	0.53	0.25	0.78	**0.64**	0.30

	Weighted	0.67	0.31	0.24	0.74	0.51	0.29	0.78	**0.52**	0.32
Combined	Binary	0.70	0.59	0.25	0.69	0.51	0.28	0.73	**0.62**	0.26
	Weighted	0.70	0.60	0.22	0.70	0.55	0.22	0.74	**0.64**	0.23

Table S4. Functional heterogeneity of modules of PPIN from Louvain algorithm, calculated for all the enriched functions. p-value = 10^{-3}

**PPIN**		**MF**			**BP**			**CC**		
		min	mean	std	min	mean	std	min	mean	std
Physical	Binary	0.06	**0.09**	0.10	0.06	0.14	0.12	0.06	0.17	0.18
	Weighted	0.05	**0.07**	0.09	0.04	0.08	0.07	0.06	0.17	0.18
Functional	Binary	0.09	0.19	0.14	0.09	**0.15**	0.11	0.09	0.23	0.14
	Weighted	0.08	0.19	0.15	**0.08**	**0.13**	0.12	0.09	0.22	0.15
Combined	Binary	0.13	**0.15**	0.08	0.13	0.15	0.08	0.13	0.23	0.17
	Weighted	0.14	0.21	0.15	0.08	**0.10**	0.06	0.09	0.21	0.16

E. Supplementary Figures

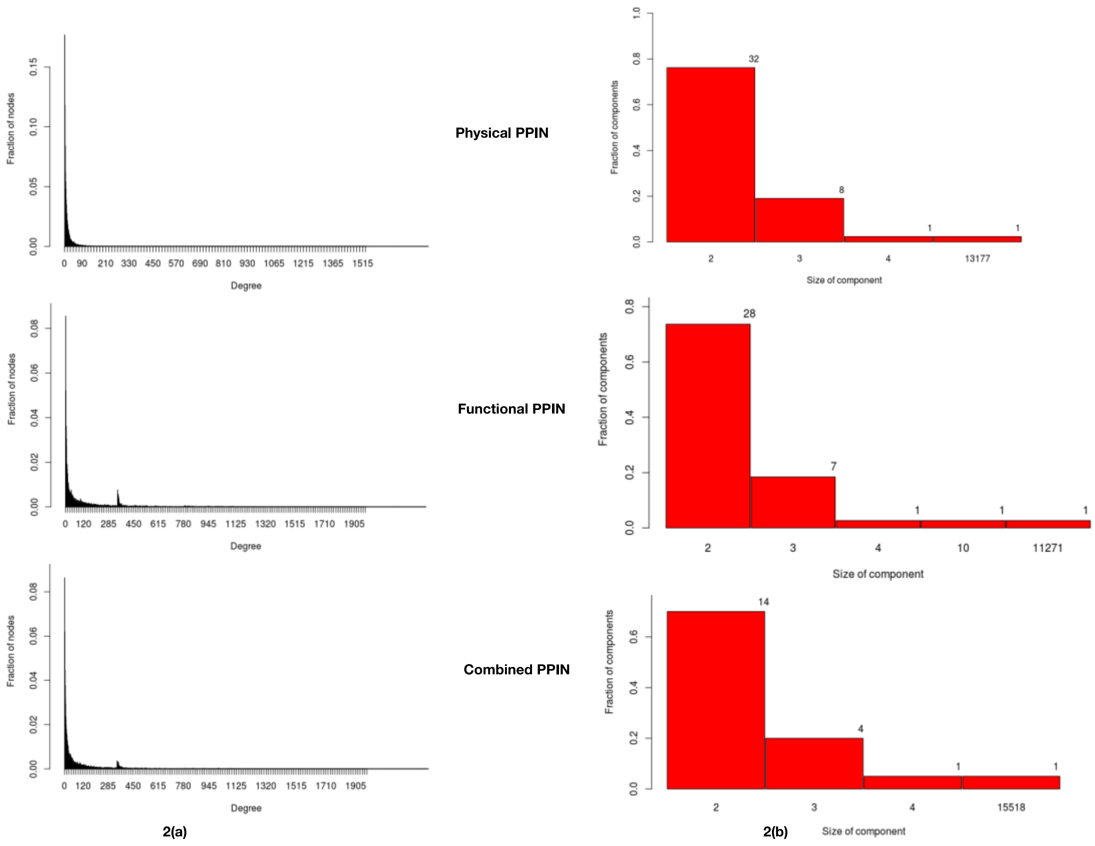

Figure S1(a) Degree distribution and (b) Connected component distribution of Physical, Functional and Combined protein-protein interaction networks.

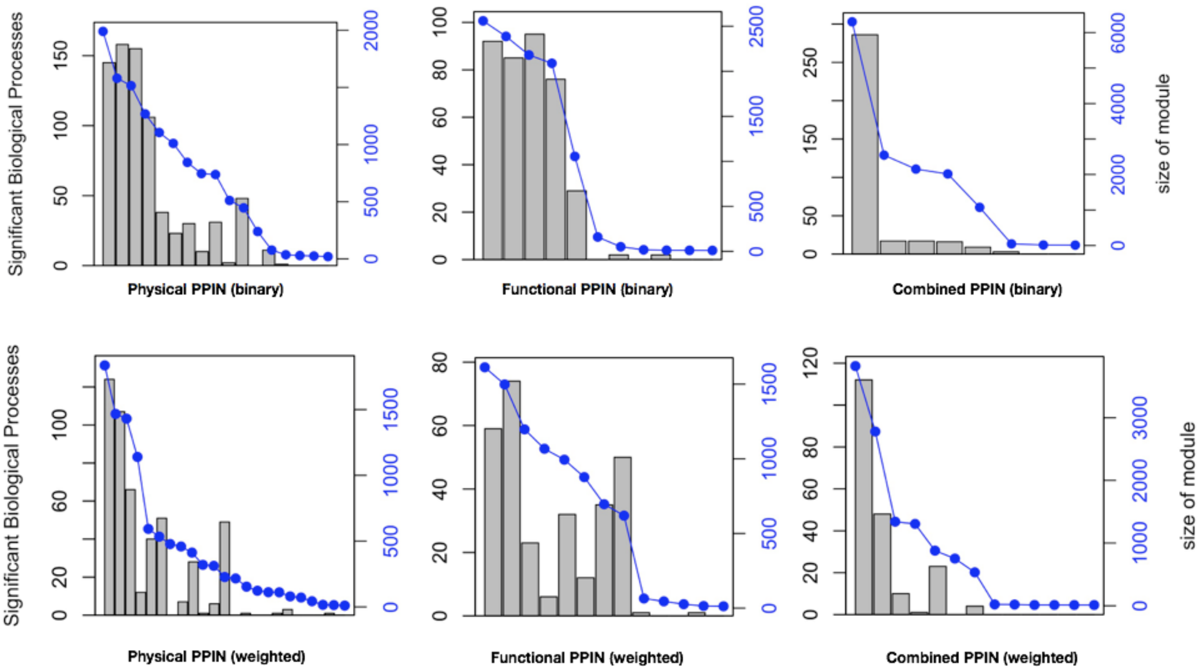

Figure S2(a). Functional enrichment analyses. Significant biological processes in Binary and Weighted networks. X-axis, y-axis (left) and y-axis (right) denotes the modules, number of statistically significant GO terms and size of modules respectively.

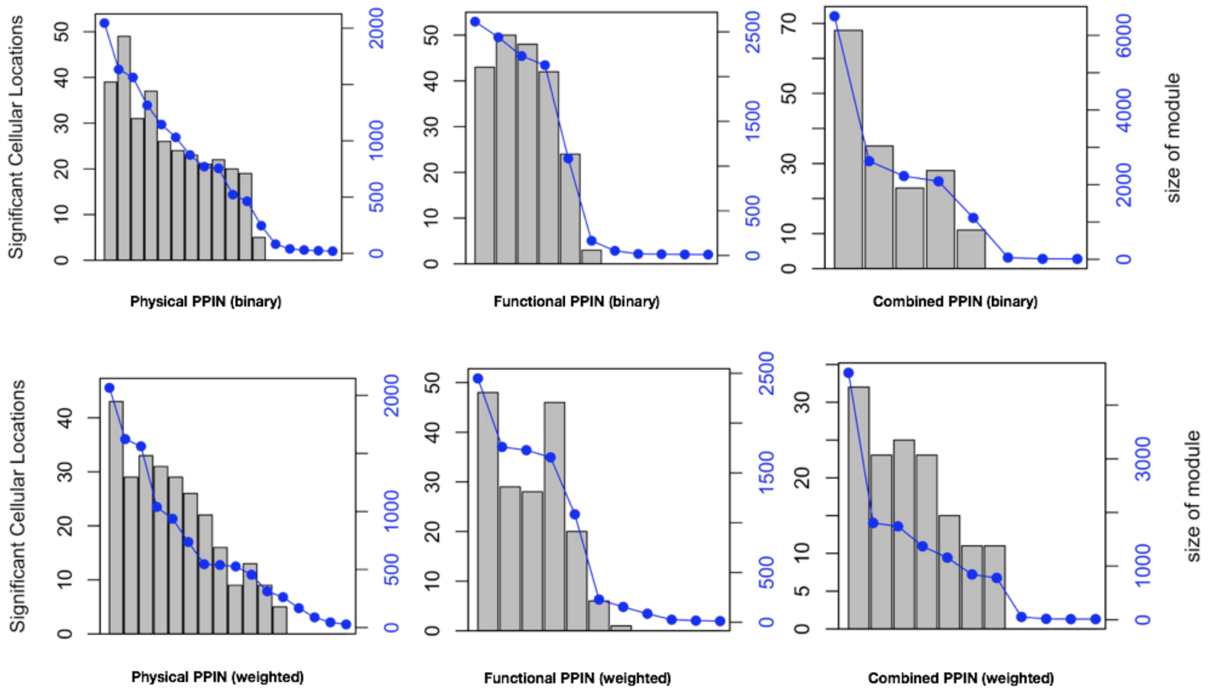

Figure S2(b). Functional enrichment analyses. Significant cellular locations in Binary and Weighted networks. X-axis, y-axis (left) and y-axis (right) denotes the modules, number of statistically significant GO terms and size of modules respectively.

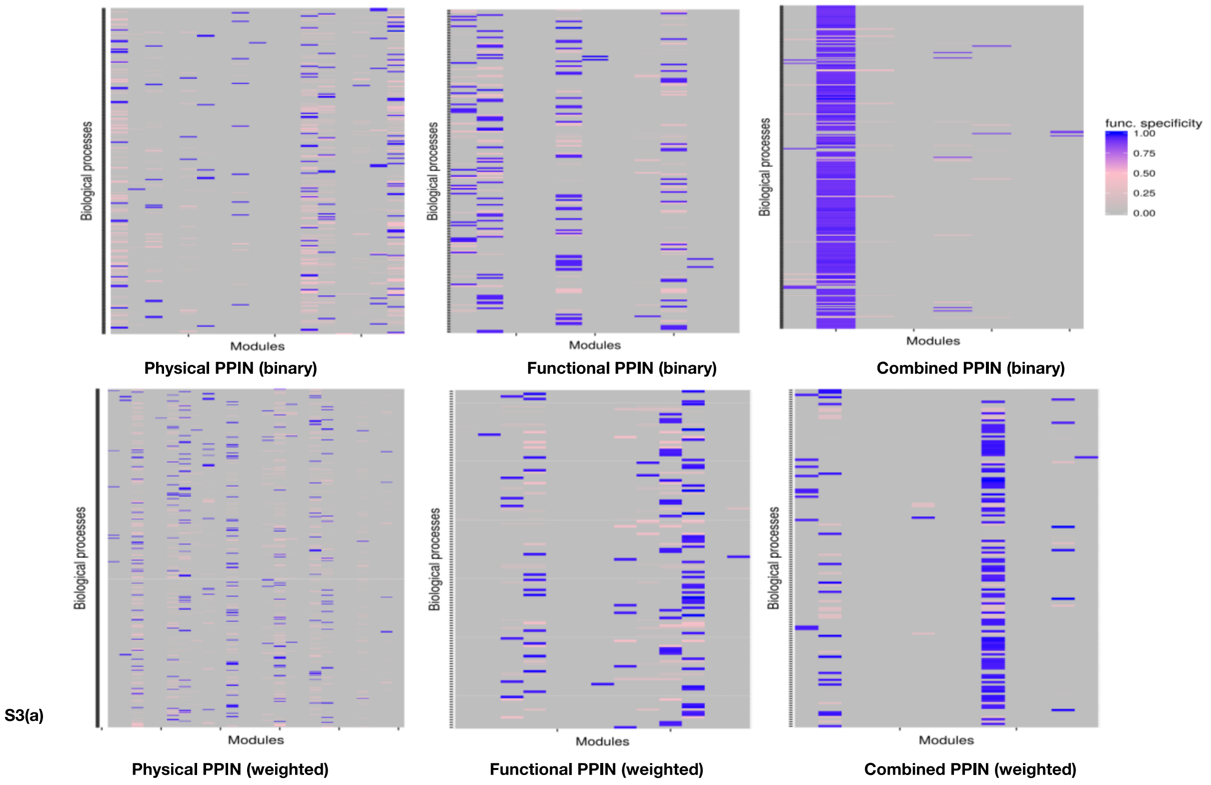

Figure S3 (a). Specificity scores s_f of topological modules for significant biological processes.

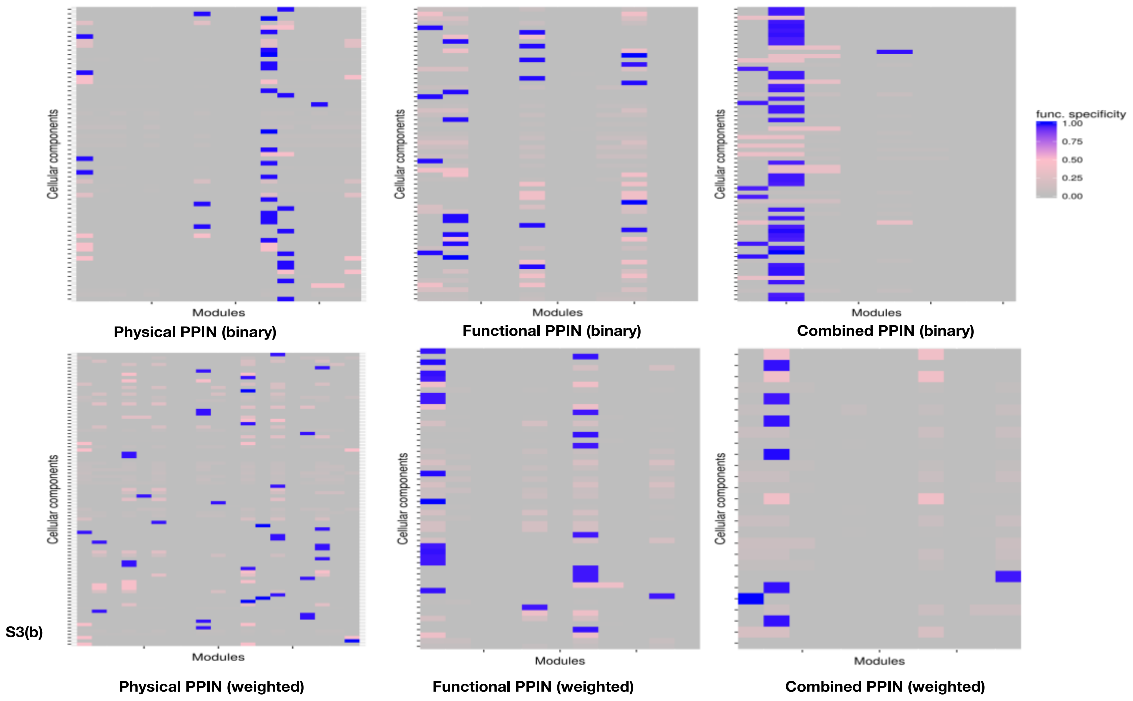

Figure S3 (b). Specificity scores s_f of topological modules for significant cellular locations.

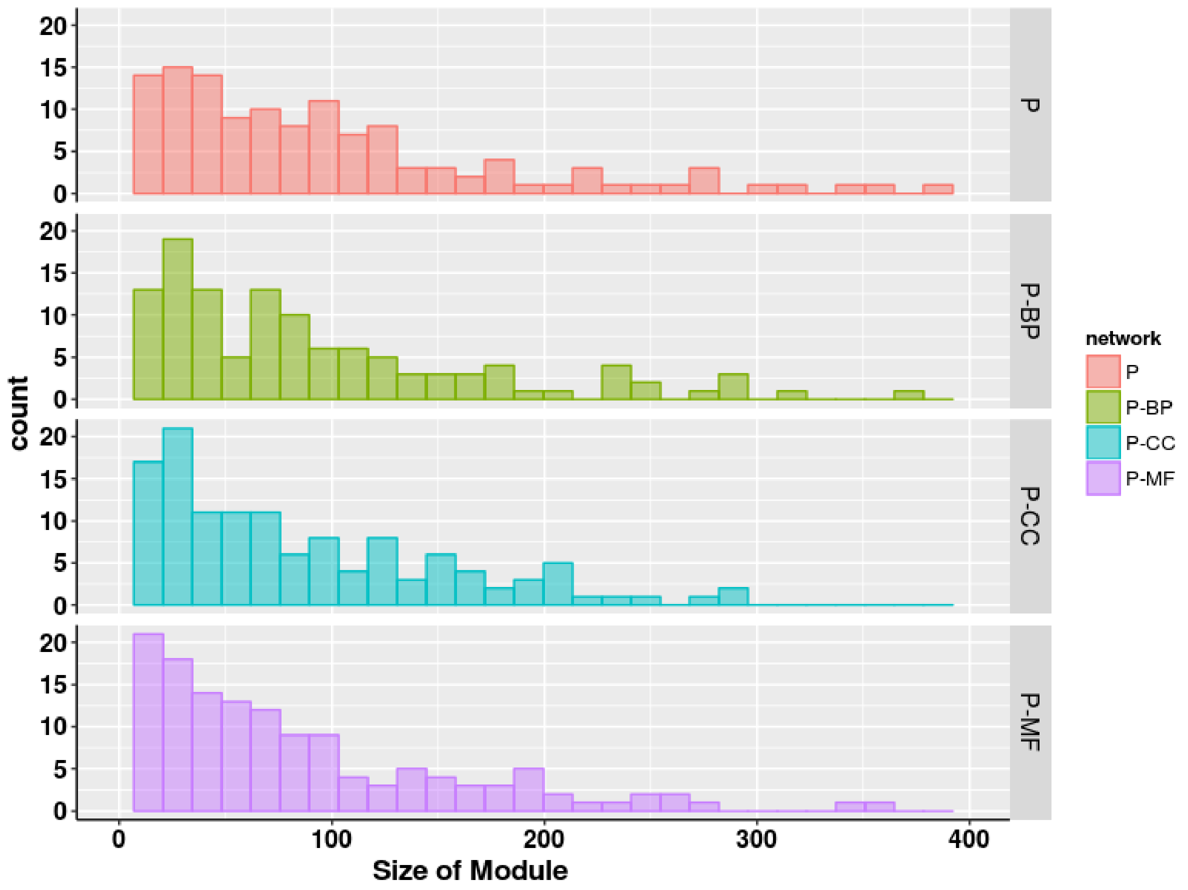

Figure S4(a). Size distributions for modules detected using Incremental Louvain algorithm in physical networks of human proteome: x-axis represents the size of modules while y-axis represents the count of meso-modules of size more than 10 nodes. P denotes the binary physical network while P-MF, P-BP and P-CC denote the weighted networks with edges scored according to functional similarity based on molecular functions (MF), biological process (BP) and cellular component (CC), respectively.

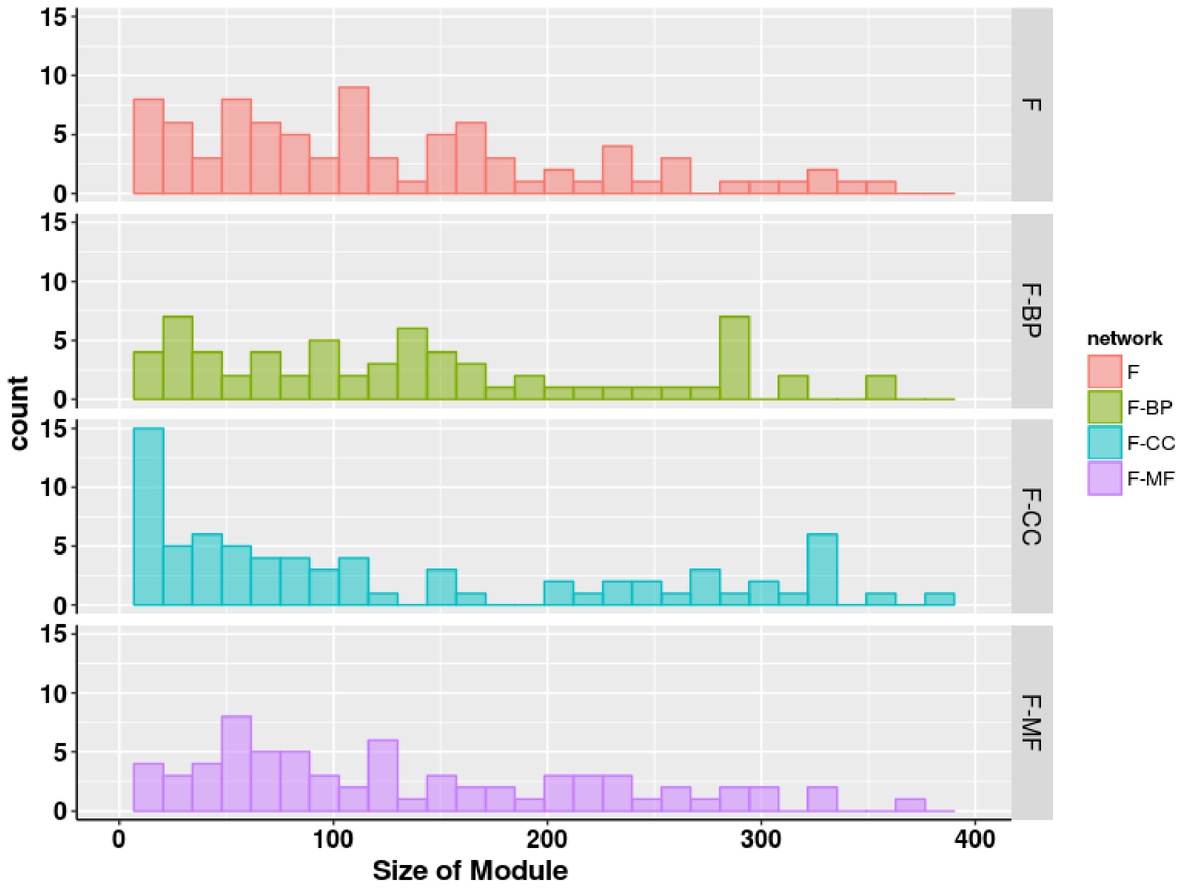

Figure S4(b). Size distributions of modules detected using Incremental Louvain algorithm in functional PPIN of human proteome. x-axis represents the size of modules while y-axis represents the count of meso-modules of size more than 10 nodes. F denotes the binary functional network while F-MF, F-BP and F-CC denote the weighted networks with edges scored according to similarity based on molecular functions (MF), biological process (BP), and cellular component (CC), respectively.

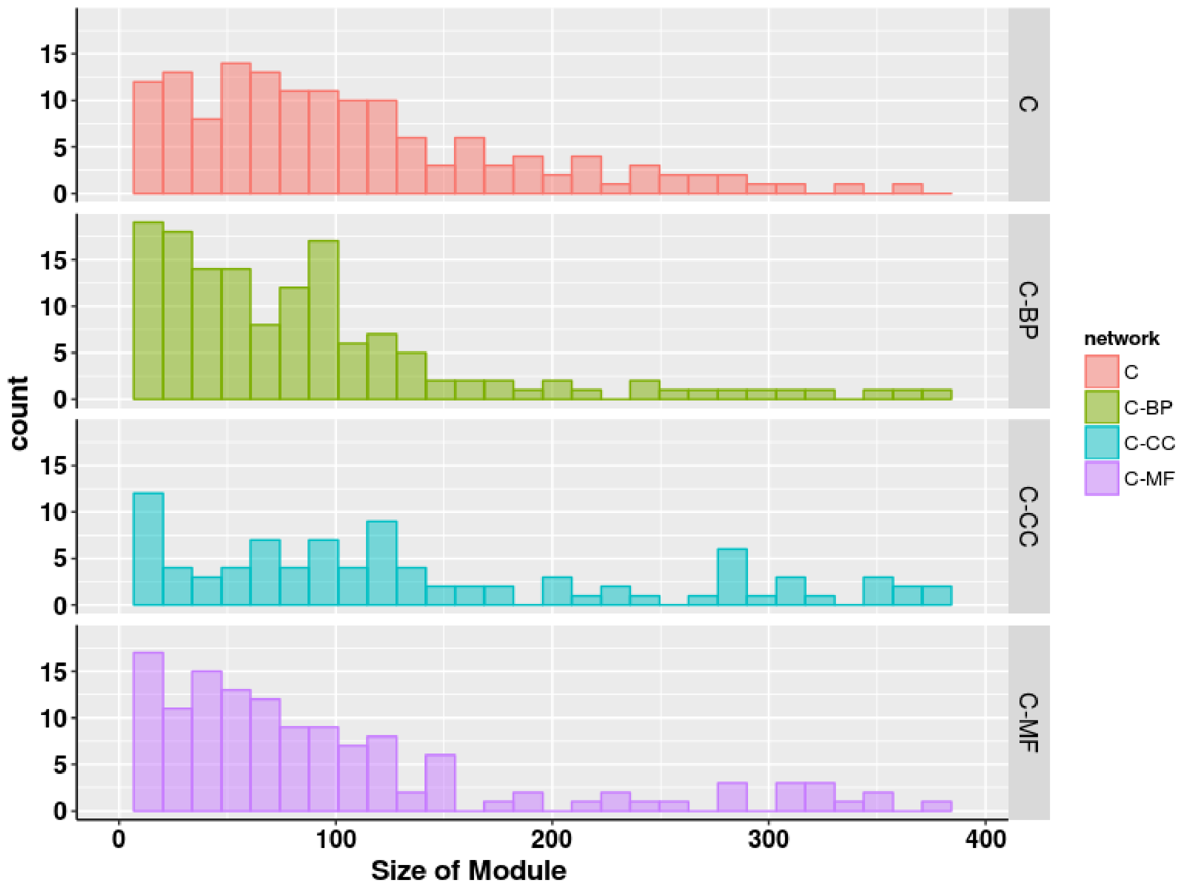

Figure S4(c). Size distributions for modules detected using Incremental Louvain algorithm in combined networks (physical and functional) of human proteome. x-axis represents the size of modules while y-axis represents the count of meso-modules of size more than 10 nodes. C denotes the combined physical network while C-MF, C-BP and C-CC denote the weighted networks where edges scored according to similarity based on molecular functions, biological process (BP), and cellular components, respectively.

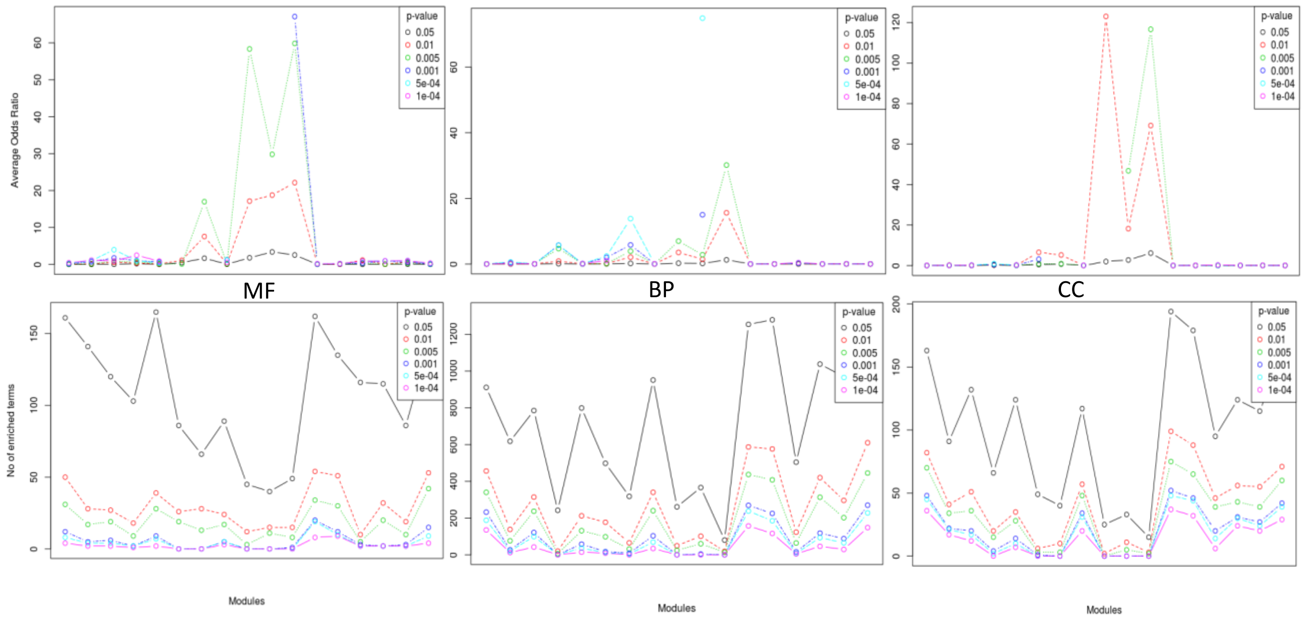

Figure S5(a). Top three figures show Average Odds-Ratio for different modules for significantly enriched GO terms from MF, BP and CC ontologies at various p-values in physical PPIN. Below three figures show number of significant GO terms from MF, BP and CC ontologies at various p-values.

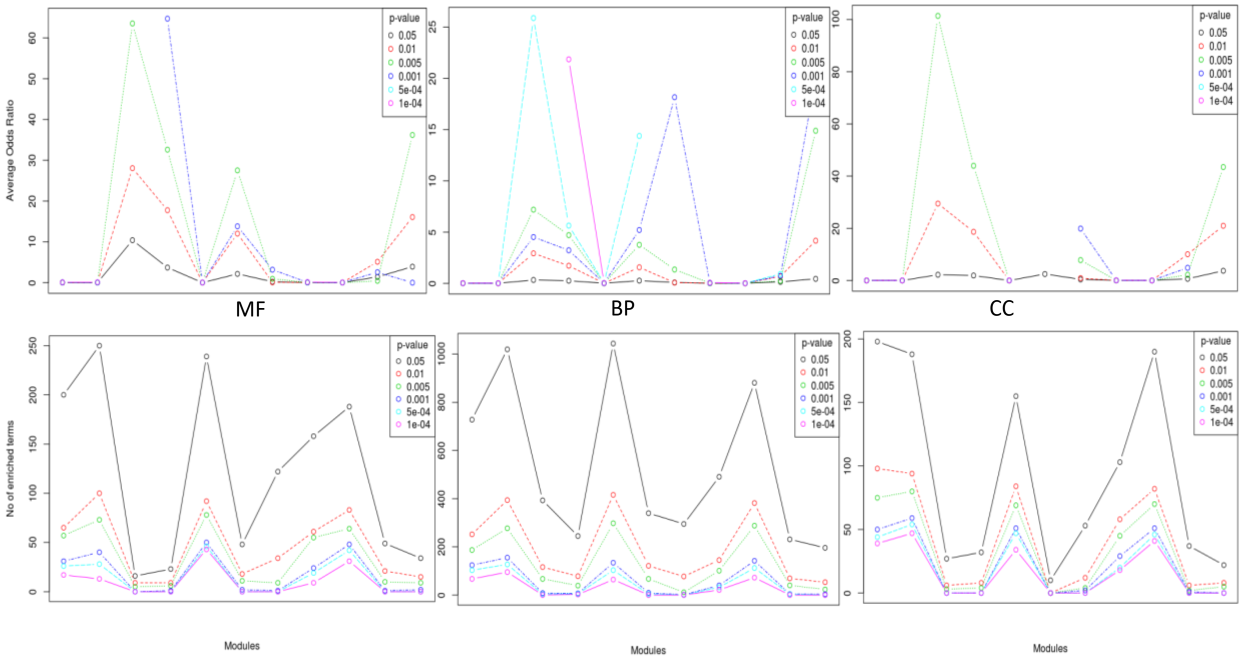

Figure S5(b). Top three figures show Average Odds-Ratio for different modules for significantly enriched GO terms from MF, BP and CC ontologies at various p-values in

functional PPIN. Below three figures show number of significant GO terms from MF, BP and CC ontologies at various p-values.

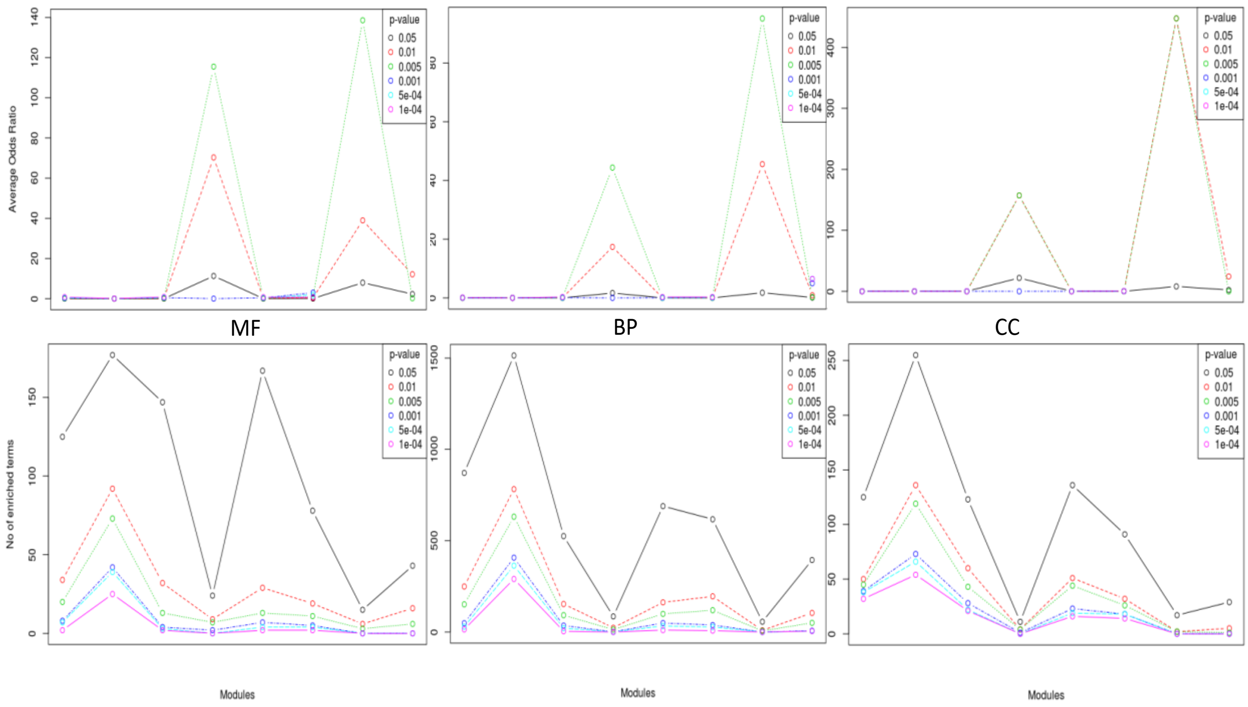

Figure S5(c). Top three figures show Average Odds-Ratio for different modules for significantly enriched GO terms from MF, BP and CC ontologies at various p-values in combined PPIN. Below three figures show number of significant GO terms from MF, BP and CC ontologies at various p-values.
